# Icosapent ethyl following acute coronary syndrome: the REDUCE-IT trial

**DOI:** 10.1093/eurheartj/ehad889

**Published:** 2024-01-22

**Authors:** Neila Sayah, Deepak L Bhatt, Michael Miller, Eliot A Brinton, Terry A Jacobson, Steven B Ketchum, Lixia Jiao, Armando Lira Pineda, Ralph T Doyle, Jean Claude Tardif, Christie M Ballantyne, Ph Gabriel Steg

**Affiliations:** Department of Cardiology, Assistance Publique–Hôpitaux de Paris, Hôpital Bichat, 46 Rue Henri Huchard, 75018 Paris, France; Mount Sinai Fuster Heart Hospital, Icahn School of Medicine at Mount Sinai Health, New York, NY, USA; Department of Medicine, Crescenz Veterans Affairs Medical Center and University of Pennsylvania School of Medicine, Philadelphia, PA, USA; Utah Lipid Center, Salt Lake City, UT, USA; Lipid Clinic and Cardiovascular Risk Reduction Program, Department of Medicine, Emory University School of Medicine, Atlanta, GA, USA; Amarin Pharma, Inc. (Amarin), Bridgewater, NJ, USA; Amarin Pharma, Inc. (Amarin), Bridgewater, NJ, USA; Amarin Pharma, Inc. (Amarin), Bridgewater, NJ, USA; Amarin Pharma, Inc. (Amarin), Bridgewater, NJ, USA; Montreal Heart Institute, Université de Montréal, Montreal, Québec, Canada; Department of Medicine, Baylor College of Medicine, and the Texas Heart Institute, Houston, TX, USA; Department of Cardiology, Assistance Publique–Hôpitaux de Paris, Hôpital Bichat, 46 Rue Henri Huchard, 75018 Paris, France; FACT (French Alliance for Cardiovascular Trials), Assistance Publique–Hôpitaux de Paris, INSERM Unité 1148, Université Paris-Cité, Hôpital Bichat, Paris, France

**Keywords:** Clinical trials, Icosapent ethyl, Acute coronary syndrome, Ischaemic events

## Introduction

The REDUCE-IT [Reduction of Cardiovascular Events With Icosapent Ethyl (IPE)-Intervention Trial] trial showed robust reductions in ischaemic events with IPE vs. placebo, including reduced cardiovascular (CV) death, albeit with increased rates of bleeding, and of atrial fibrillation (AF).^1^ These observations were confirmed in patients with prior myocardial infarction (MI).^2^ Patients with recent (<12 months) acute coronary syndrome (ACS) are at very high risk of future CV events (including arrhythmias) for which they usually receive intensive antithrombotic therapy, which might increase bleeding risk with IPE. The benefit and safety of IPE in this specific patient subgroup are largely unknown and were explored in the present *post hoc* analysis of the original trial database.

## Methods

REDUCE-IT was a double-blind, placebo-controlled trial that randomized 8179 statin-treated patients with controlled low-density lipoprotein cholesterol and moderately elevated triglycerides, and with either established CV disease or with diabetes and at least one additional risk factor, to either 4 g IPE or placebo.^1^ The trial methods and results have previously been published.^1,3^ The primary outcome was a composite of CV death, non-fatal MI, non-fatal stroke, coronary revascularization, or hospitalization for unstable angina. The key secondary outcome was a composite of CV death, non-fatal MI, or non-fatal stroke.

Recent ACS (*n* = 840) was defined as MI or unstable angina within 12 months before randomization. In subsequent analyses, this group was compared with patients experiencing ACS ≥12 months before randomization (*n* = 3651). Time-to-first event was analysed by Kaplan–Meier analysis and compared using the log-rank test. Hazard ratios (HRs) and 95% confidence intervals (CIs) were generated using a Cox proportional hazards model computed to determine the risk of primary and secondary outcomes according to the use of IPE vs. placebo in two-sided analyses. The model was stratified by the three randomization factors of CV risk category (established CV disease or diabetes plus risk factors), geographic region, and baseline ezetimibe use. Time-to-subsequent events was analysed using the Wei, Lin, and Weissfeld model to estimate HR and 95% CI for treatment effects. Total events were analysed by negative-binomial regression model to estimate rate ratio (RR) and 95% CI for treatment effects. Absolute risk reduction (ARR) was calculated as difference of event incidence rates between IPE and placebo. Statistical analyses were performed using SAS version 9.4 software (SAS Institute, Inc.).

## Results

Among 8179 patients in REDUCE-IT, there were 840 (10.3%) with recent ACS <12 months before randomization. Median age was 59.5 years, 646 (76.9%) were male, 310 (36.9%) had diabetes, 839 (99.9%) were on statins, 805 (95.8%) received antiplatelet therapy, 584 (69.5%) received dual antiplatelet therapy (DAPT), 54 (6.4%) were on oral anticoagulant therapy, and 39 (4.6%) were on anticoagulant plus antiplatelet therapy. Median baseline triglyceride levels were 219.5 (interquartile range (IQR) 180.5–272.5) and 213.0 (IQR 174.0–274.0) mg/dL in the IPE and placebo groups, respectively (*P* = .36).

The median time elapsed between the index ACS and randomization was similar between IPE and placebo arms {5.5 (IQR 3.0–8.1) vs. 5.6 (IQR 3.2–8.4) months; *P* = .63}. Among recent ACS patients, use of anti-diabetic, anti-hypertensive, angiotensin-converting enzyme inhibitor, or beta-blocker therapies was similar between treatment arms, but DAPT use was lower in the IPE group compared with the placebo group (66.3% vs. 73.0%, *P* = .04), and rates of anticoagulant plus antiplatelet therapy were higher in the IPE group (6.2% vs. 2.9%, *P* = .02). Median study follow-up time was 4.75 years. Maximum follow-up time was 5.94 years.

**Figure 1:**
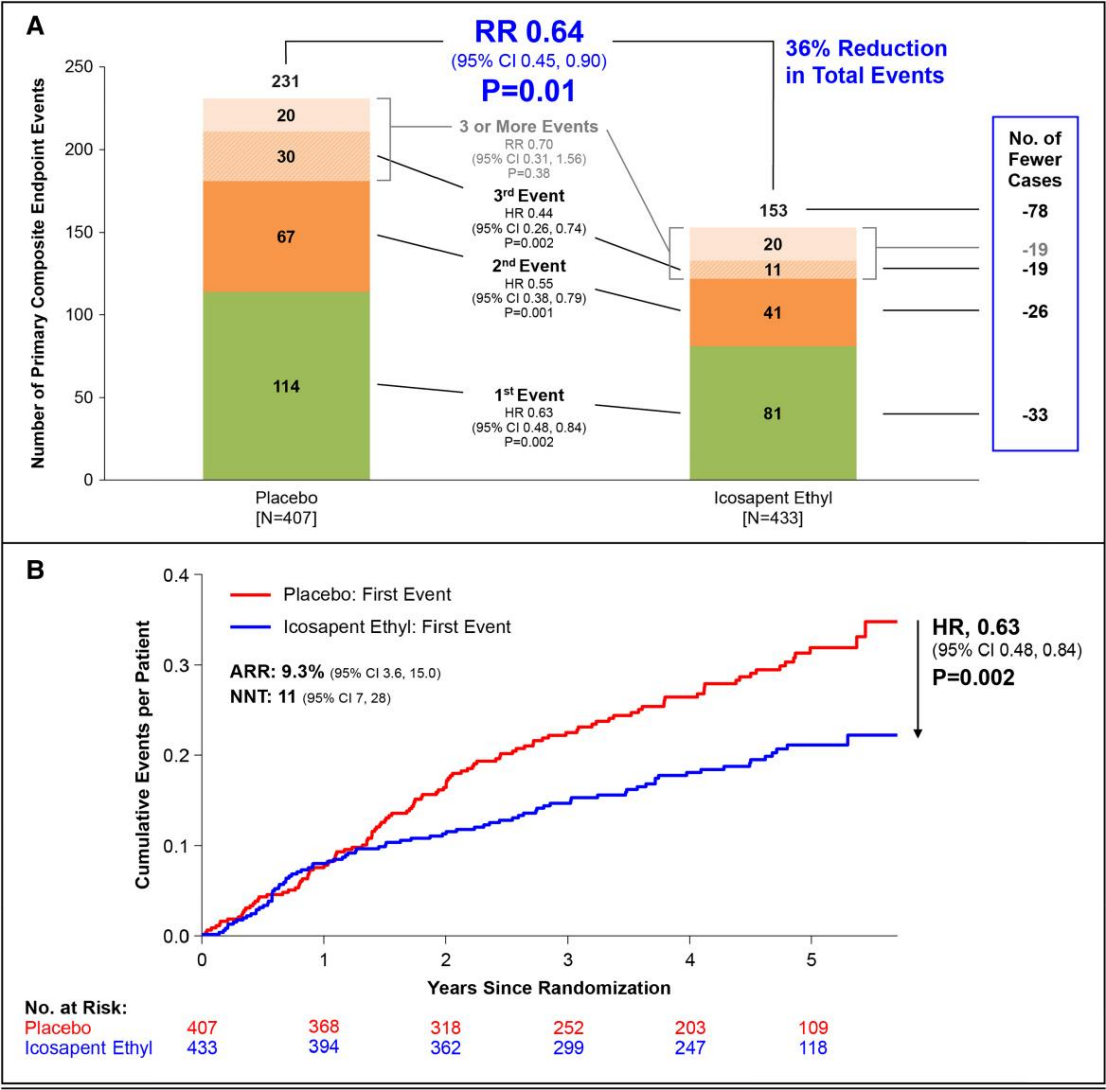
(*A*) First and subsequent events for the primary composite outcome in patients with recent ACS. (*B*) Cumulative incidence curves of the primary composite outcome in patients with recent ACS

Icosapent ethyl reduced the incidence of the first primary composite outcome by 37% (HR 0.63; 95% CI 0.48–0.84, *P* = .002) and total primary composite outcomes by 36% (RR 0.64; 95% CI 0.45–0.90, *P* = .01) (*Figure 1*). Absolute risk reduction in first primary outcome with IPE was 9.3%, (18.7% vs. 28.0%), number needed to treat (NNT) being 11 (95% CI 7–28). In contrast, these benefits tended to be less among the 3651 patients with ACS ≥12 months before randomization, in whom the relative risk reduction (RRR) was 22.0% and the ARR was 4.7% (NNT = 21) (HR 0.78; 95% CI 0.68–0.90, *P* = .0004) (interaction between recent and non-recent ACS *P* = .17). In recent ACS patients, IPE reduced the incidence of the first key secondary composite outcome by 36% (HR 0.64; 95% CI 0.44–0.92, *P* = .01) and total key secondary composite outcomes by 28% (RR 0.72; 95% CI 0.47–1.09, *P* = .12).

In recent ACS patients, IPE lowered the composite of CV death and non-fatal MI 36% (HR 0.64; 95% CI 0.43–0.96, *P* = .03) with an ARR of 5.0%, and lowered urgent or emergent revascularization 44% (HR 0.56; 95% CI 0.36–0.87, *P* = .009) with an ARR of 5.1%.

In contrast, among recent ACS patients, the proportion of patients with at least one treatment-emergent adverse event (TEAE) did not differ between treatment arms (78.8% for IPE vs. 76.7% for placebo, Fisher’s exact *P* = .51), nor did total bleeding or bleeding-related serious adverse events (6.9% vs. 8.1%, Fisher’s exact *P* = .60 and 1.6% vs. 3.2%, Fisher’s exact *P* = .17, respectively). Among recent ACS patients who were on DAPT at study entry (*n* = 584), the percentage with at least one treatment-emergent bleeding adverse event was 7.7% in the IPE arm and 9.4% with placebo (Fisher's exact *P* = .46). No haemorrhagic strokes occurred in either arm. Similar to the overall trial, among recent ACS patients, TEAEs of AF or flutter were higher in the IPE arm than with placebo (7.4% vs. 2.9%, Fisher's exact *P* = .005), as was the safety endpoint of hospitalizations for AF or flutter (4.8% vs. 1.7%, log-rank *P* = .01).

## Discussion

In REDUCE-IT patients with recent ACS (<12 months before randomization), IPE dramatically reduced ischaemic CV events compared with placebo, apparently more dramatically than in the overall trial (ARR of 9.3%, NNT of 11 vs. ARR 4.8%, NNT of 21).^1^ This benefit accrued without increased bleeding, even in patients receiving DAPT. The rates of urgent or emergent revascularization were also reduced, consistent with the results in the overall REDUCE-IT population and in the subgroup with prior MI.^4–8^ On the other hand, the risk of AF or flutter was increased with IPE, as previously reported in REDUCE-IT^1,2^ and in other trials of omega 3 fatty acids,^9,10^ but with no increased risk of stroke. Given the *post hoc* nature of this analysis and the lack of adjustment for multiplicity, these results should be interpreted with caution and require further confirmation. However, these findings reaffirm the importance of targeting high-risk patients to achieve substantial benefit and the importance of targeting patients with elevated triglycerides early after ACS (within 12 months after the index event).

## Conclusion

In this *post hoc* subgroup analysis of REDUCE-IT, IPE dramatically reduced the risk of ischaemic events in high-risk, statin-treated patients with recent ACS (<12 months), without excess bleeding. This supports initiation of IPE in REDUCE-IT-eligible patients as soon as possible after ACS.

## Data Availability

The data underlying this article will be shared on reasonable request to the corresponding author.

## Pre-registered Clinical Trial Number

The registered clinical trial number is NCT01492361.
